# Prevalence and predictors of psychological distress following injury: findings from a prospective cohort study

**DOI:** 10.1186/s40621-021-00337-7

**Published:** 2021-06-21

**Authors:** Amy E. Richardson, Sarah Derrett, Ariyapala Samaranayaka, Emma H. Wyeth

**Affiliations:** 1 Injury Prevention Research Unit, Department of Preventive and Social Medicine, Dunedin School of Medicine, University of Otago, PO Box 56, Dunedin, 9054 New Zealand; 2 Biostatistics Centre, Division of Health Sciences, University of Otago, PO Box 56, Dunedin, 9054 New Zealand; 3 Ngāi Tahu Māori Health Research Unit, Department of Preventive and Social Medicine, Dunedin School of Medicine, University of Otago, PO Box 56, Dunedin, 9054 New Zealand

**Keywords:** Injury, Psychological distress, Mental health, Injury outcomes, Predictors, Income, Healthcare access

## Abstract

**Background:**

Research examining psychological distress in people who have experienced an injury has focused on those with serious injuries or specific injury types, and has not involved long-term follow up. The aims of this investigation were to describe the prevalence of, and factors contributing to, psychological distress in a cohort of people with a broad range of injuries.

**Methods:**

The Prospective Outcomes of Injury Study (POIS) is a longitudinal cohort study of 2856 injured New Zealanders recruited from a national insurance entitlement claims register between 2007 and 2009. Participants were interviewed approximately 3, 12, and 24 months after their injury. The Kessler Psychological Distress Scale (K6) was used to measure psychological distress at each interview.

**Results:**

25% of participants reported clinically relevant distress (K6 ≥ 8) 3 months post-injury, 15% reported distress at 12 months, and 16% reported distress at 24 months. Being 45 years or older, Māori or Pacific ethnicity, experiencing pre-injury mental health conditions, having inadequate pre-injury income, reporting poor pre-injury health or trouble accessing healthcare, having a severe injury or an injury resulting from assault, and reporting clinically relevant distress 3 months post-injury were independently associated with an increased risk of distress 12 months post-injury. The majority of these associations were also evident with respect to distress 24 months post-injury.

**Conclusions:**

Distress is common after injury among people with a broad range of injury types and severities. Screening for distress early after injury is important to identify individuals in need of targeted support.

## Background

Globally, injury is prevalent and an important contributor to ill health and mortality (Kmietowicz 2015). In 2013, injuries accounted for 10% of the global disability burden, with 973 million people sustaining injuries that required medical care (Haagsma et al. 2016). Understanding factors influencing recovery from injury is important to improve the provision of targeted healthcare interventions and support for injured people.

A key factor influencing post-injury health status is psychological distress (Richmond et al. 2014). Psychological distress is a broad concept used to describe a state of emotional suffering that interferes with a person’s functioning, and is typically characterised by symptoms of anxiety and depression (Pratt 2009). Evidence indicates that distress is common post-injury (Mason et al. 2002). In Australia, a study of 201 trauma centre patients interviewed when hospitalised, and again 3 and 6 months later, found that over half of patients reported higher than average levels of depression, anxiety or stress in at least one interview (Wiseman et al. 2015). Among 4883 patients hospitalised for injury in the Netherlands, symptoms of depression, anxiety, and post-traumatic stress were common one week post-injury and rates had reduced only slightly by 24 months post-injury (Kruithof et al. 2020).

In addition to distress symptoms, serious psychiatric disorders have been found to occur after injury. In a prospective cohort study of 1084 traumatically injured patients in Australia, 31% had a psychiatric disorder 12 months after injury; 22% of whom had never experienced the disorder before (Bryant et al. 2010). New disorders included depression, generalised anxiety disorder, post-traumatic stress disorder and agoraphobia. A similar prevalence of psychiatric morbidity has been identified even after excluding individuals with traumatic brain injury (O’Donnell et al. 2004).

Interestingly, there is little evidence to suggest that severity of a physical injury is associated with severity of psychological distress post-injury (Chiu et al. 2011), although individual perceptions of injury are important (Brasel et al. 2010). In the United States, a study of 248 patients seeking emergency care for a minor injury found that 18% were diagnosed with depression 12 months after their injury (Richmond et al. 2009). These patients were found to be less likely to return to pre-injury levels of function, work status, and health compared to their non-depressed counterparts (Richmond et al. 2009), and experienced a significant reduction in quality of life (Richmond et al. 2014). Depression and anxiety early after injury have also been associated with clinically relevant reductions in health-related quality of life outcomes up to 12 months later, among a cohort of 668 patients hospitalised for injury in the United Kingdom (Kendrick et al. 2017).

Despite the significant influence of psychological distress on a range of health outcomes post-injury (Kellezi et al. 2017), there has been limited investigation of the factors contributing to the occurrence of psychological distress in injured populations. Studies that have been conducted have been restricted to specific injury types (e.g. traumatic brain injury) (Andruszkow et al. 2014) or sub-groups (e.g. motor vehicle injuries) (Ehring et al. 2006). Studies that have examined predictors of psychological distress in general injury populations have focused exclusively on individuals requiring hospital care for their injury and have investigated a limited range of predictors (Chiu et al. 2011; de Munter et al. 2020; Richmond and Kauder 2000; Shalev et al. 2019; Wiseman et al. 2015). In the study of 201 Australian trauma patients, intensive care unit admission and high levels of depression, anxiety and stress at 3 months post injury were predictors of high levels of depression, anxiety and stress at 6 months (Wiseman et al. 2015). Elsewhere, pre-injury frailty, psychological complaints, and non-working status pre-injury, female sex, low educational level, and road traffic injury were identified as prognostic of anxiety, depression, or post-traumatic stress symptoms among 4239 patients from 10 hospitals in the Netherlands (de Munter et al. 2020).

While existing research provides important insights, it is unclear whether factors predictive of distress among hospitalised injured people can be generalised to those with injuries treated within primary care settings. Such injuries are traditionally viewed as ‘minor’ (in terms of short-term ‘threat to life’) yet represent the vast majority of injuries (Polinder et al. 2012), and account for more than two thirds of years lived with disability after injury (Lyons et al. 2011). Given the significant psychological distress observed among people who experience a minor injury, and the implications for their subsequent quality of life and functioning (Richmond et al. 2014; Richmond et al., 2009), it is important to identify factors that contribute to distress in this group. By doing so, screening tools to identify individuals at risk of experiencing psychological distress after injury can be developed and preventive interventions can be implemented.

Using data from the Prospective Outcomes of Injury Study (POIS) (Derrett et al. 2009), a longitudinal cohort study of 2856 New Zealanders with a diverse range of injuries (Derrett et al. 2011), this investigation aims to: 1) describe the prevalence of psychological distress among POIS participants at 3, 12, and 24 months post-injury; and 2) identify pre- and early post-injury factors contributing to clinically relevant distress up to 24 months post-injury.

## Methods

### Participants

Detailed information on the design of POIS has been reported previously (Derrett et al. 2011). Participants were randomly selected from the entitlement claims register of the Accident Compensation Corporation (ACC), New Zealand’s no-fault injury compensation scheme funded by the government (and government prescribed levies). People on this register are eligible for support to help them recover from their injury, such as rehabilitation and treatment costs, home help, and compensation for lost wages. Claims are lodged by health professionals on behalf of injured individuals following hospitalisation, emergency department presentation, or consultation with other ACC-approved health professionals (e.g. general practitioners, physiotherapists).

Participants were recruited, following an acute injury event, from five regions throughout New Zealand (Auckland City, Manukau City, Gisborne, Otago, and Southland) between late-2007 and mid-2009. Duration of recruitment was extended to ensure adequate representation of Māori (Derrett et al. 2011), the indigenous people of New Zealand (NZ). Participants were aged 18 to 64 years at the time of their injury. All injury types were eligible except those resulting from sexual assault or self-harm.

### Study design

POIS was designed to identify predictors of health, wellbeing, and disability outcomes following injury (Derrett et al. 2011). Participants completed interviews at approximately 3, 12, and 24 months post-injury, with interviews collecting information on a range of pre-injury, injury-related and post-injury factors. Ethical approval was obtained from the New Zealand Health and Disability Multi-region Ethics Committee (MEC/07/07/093).

### Outcome measure

The Kessler Psychological Distress Scale (K6) was used to measure psychological distress (Kessler et al. 2003). This widely used measure is comprised of six items designed to screen for serious mental illness in the general population (Kessler et al. 2010). Items ask respondents to indicate distress symptoms over the past 30 days using a 5-point scale, with response options ranging from ‘0=none of the time’ to ‘4=all of the time’. Items are summed to calculate a total score between 0 and 24. Scores can be categorised into three groups: 0–7 representing probable absence of mental illness, 8–12 probable mild-moderate mental illness, and ≥ 13 probable serious mental illness (Wang et al. 2007). Scores ≥8 indicate a clinically relevant level of distress that warrants mental health intervention (Prochaska et al. 2012). The K6 has demonstrated high accuracy at discriminating cases of clinically relevant distress from non-cases, as well as excellent psychometric properties (validity, reliability, and sensitivity) across diverse populations (Kessler et al. 2002; Kessler et al. 2010).

### Explanatory variables

As in previous POIS analyses, explanatory variables were grouped into four dimensions: pre-injury, injury-related, health service-related, and early post-injury characteristics.

#### Pre-injury characteristics

At the first interview, participants were asked about a range of pre-injury sociodemographic, socioeconomic, and health-related characteristics. Questions from the New Zealand Census (Statistics New Zealand 2006) were used to collect information about age, sex, ethnicity (prioritised in accordance with Statistics NZ standards), living arrangements (classified as ‘living alone or with non-family’ and ‘living with family, including partner/spouse’), and highest educational qualification (classified as ‘less than secondary school’ and ‘secondary school or higher’). Participants were asked about the adequacy of their household income to meet everyday needs (classified as ‘adequate’ if participants reported having ‘more than enough’ or ‘enough’, and ‘inadequate’ if participants reported ‘just enough’ or ‘not enough’) (Ministry of Social Development 2000; Derrett et al. 2011), and whether they were working for pay before their injury (classified as ‘yes’ if working full or part-time and ‘no’ if not). With respect to pre-injury health, participants were asked whether they had previously been told by a doctor that they had one or more of a list of 21 long-term health conditions (4 mental and 17 physical conditions; e.g. depression, anxiety, asthma, cancer or diabetes) that had lasted, or were expected to last, for more than 6 months (Ministry of Health 2008). Participants also rated their pre-injury general health using a 5-point scale; responses were grouped as ‘excellent/very good’, ‘good’, and ‘fair/poor’ (Ware et al. 2000). Pre-injury alcohol use was assessed with the AUDIT-C and classified as ‘hazardous’ and ‘non-hazardous’ (Bush et al. 1998).

#### Injury-related characteristics

Injury severity was assessed using a derived New Injury Severity Score (NISS) (Lavoie et al. 2004) grouped into NISS 1–3 (least severe), 4–6 (severe) and > 6 (most severe). Participants were asked whether their injury was intentional or not (‘assault’ or ‘unintentional’) and whether at the time of their injury they thought the injury presented a threat to their life or of severe long-term disability (‘yes’ or ‘no’).

#### Health service-related characteristics

Participants were also asked about their experience of healthcare services for the treatment and management of their injury, and their experience of contact with ACC, with possible response options for both questions of ‘very good’, ‘good’, ‘moderate’, ‘bad’, and ‘very bad’. A question about trouble getting to or accessing health services for their injury was also asked of participants; responses were classified as ‘trouble/mixed’ and ‘no trouble’.

#### Early post-injury characteristics

K6 scores at the 3-month interview were investigated as a potential explanatory variable contributing to distress at subsequent interview points (12 and 24 months). Expectations of future recovery from injury were also evaluated at 3 months with participants asked whether their injury was still affecting them (‘yes’ or ‘no, I have completely recovered’) and if so, whether they thought they would get ‘better soon’, ‘better slowly’ (classified as ‘better soon/slowly’), ‘do not know’ or ‘never get better’. Satisfaction with social relationships (‘completely satisfied’ and ‘mostly satisfied’ classified as ‘satisfied’ and ‘neither satisfied nor dissatisfied’, ‘mostly dissatisfied’, and ‘completely dissatisfied’ classified as ‘mixed/dissatisfied’) was also reported.

### Statistical analyses

Analyses were conducted using StataIC 16 (StataCorp 2017). Descriptive statistics were performed to describe the characteristics of participants reporting clinically relevant distress (K6 scores ≥8) at each interview. Associations between each explanatory variable and distress at 12 months and 24 months post-injury were examined using univariate modified Poisson regression (Zou 2004). Next, multivariable modified Poisson regression models (Zou 2004) were developed to identify variables (pre-injury, injury-related, health service-related, and early post-injury) associated with distress at 12 months post-injury after accounting for other variables, and to see whether these variables continued to be associated with distress 24 months post-injury. A stepwise backward selection algorithm with a *P*-value threshold of ≤0.10 was used to identify variables to retain in each multivariable model (to safeguard against eliminating variables of marginal statistical significance), and the results of the univariate analyses.

## Results

Of 2856 participants recruited to POIS, 2821 had K6 outcome data available at 3 months (99%), 2239 at 12 months (78%), and 2217 at 24 months (78%). Table 1 presents the number of participants with probable mild-moderate mental illness and probable serious mental illness, respectively, at each interview time point.

**Table 1:** Number of participants reporting clinically relevant distress at 3, 12, and 24 month interviews

	3 Months *n* = 2821	12 Months *n* = 2239	24 Months *n* = 2217
Mild-moderate distress			
Yes	699 (25%)	333 (15%)	353 (16%)
No	2122 (75%)	1906 (85%)	1864 (84%)
Probable serious distress			
Yes	235 (8%)	84 (4%)	94 (4%)
No	2586 (92%)	2155 (96%)	2123 (96%)

The pre-injury, injury, health service-related, and early post-injury characteristics of participants reporting distress likely to be clinically relevant (K6 scores ≥8) at each interview are displayed in Table 2.

**Table 2:** Pre-injury, injury, and early post-injury characteristics of distressed and non-distressed participants at each interview

	3 Months		12 Months		24 Months	
	Not Distressed (*n* = 2122)	Distressed (*n* = 699)	Not Distressed (*n* = 1906)	Distressed (*n* = 333)	Not distressed (*n* = 1864)	Distressed (*n* = 353)
Pre-Injury Characteristics						
*Sex*						
Male	1308	421	1135	182	1105	193
Female	814	278	771	151	759	160
*Age (Years)*						
18–24	296	106	235	36	212	41
25–34	431	159	378	59	367	73
35–44	467	165	436	72	425	81
45–54	515	177	488	104	483	101
55–65	413	92	369	62	377	57
*Ethnicity (Prioritised)*						
European	1208	328	1144	155	1147	167
Māori	387	170	308	91	302	75
Pacific	124	76	94	28	89	32
Asian	156	56	143	22	115	36
Other	241	69	215	37	209	42
*Education*						
Less than secondary school	498	231	445	122	430	113
Secondary school or higher	1574	456	1430	207	1402	235
*Living Arrangements*						
Alone/With non-family	384	139	315	70	317	67
With family	1727	556	1584	263	1539	286
*Income Adequacy*						
Adequate	1381	392	1278	154	1281	167
Inadequate	717	299	614	177	567	182
*Working For Pay*						
No	160	67	151	34	135	34
Yes	1962	631	1755	299	1729	319
*Mental Health Conditions*						
0	1878	546	1686	246	1640	267
1	123	72	107	52	113	44
2+	47	61	59	33	48	37
*Physical Health Conditions*						
0	1168	350	1050	154	1009	169
1	588	187	524	89	518	99
2+	292	142	278	88	274	80
*General Health*						
Excellent/Very good	1504	441	1362	177	1337	195
Good	516	197	465	110	445	112
Fair/poor	102	57	77	44	77	44
*Alcohol Use*						
Non-hazardous	722	229	658	118	642	142
Hazardous	1376	465	1233	213	1206	211
Injury-Related Characteristics						
*Injury Severity (NISS)*						
1–3 (Least severe)	930	275	768	159	755	167
4–6 (Severe)	968	320	917	129	878	147
7+ (Most Severe)	199	96	195	44	205	37
*Cause of Injury*						
Unintentional	2059	639	1852	302	1803	328
Intentional (Assault)	57	54	49	29	53	23
*Perceived Threat to Life*						
No	1914	532	1704	258	1654	281
Yes	188	142	176	67	182	67
Health Service Characteristics						
*Health Service Experience*						
Very good/Good/Moderate	2051	655	1842	315	1803	327
Bad/Very bad	61	39	54	17	52	24
*ACC Experience*						
Very good/Good/Moderate	1884	612	1713	296	1669	302
Bad/Very bad	108	52	101	22	98	29
*Access to Health Services*						
No Trouble	1914	600	1722	280	1679	298
Trouble/Mixed	191	91	168	46	166	51
Early Post-Injury Characteristics						
*Distress at 3 Months (K6 ≥ 8)*						
Not Distressed	2122	0	1553	135	1505	156
Distressed	0	699	335	191	343	190
*Expectations for Recovery*						
Already Recovered	587	53	452	46	429	50
Expect to Recover Soon/Slowly	1175	425	1114	180	1091	190
Unsure	244	170	245	77	241	85
Expect to Never Recover	66	28	58	18	60	15
*Satisfaction with Social Relationships*						
Satisfied	1946	426	1681	228	1635	244
Neutral/ Dissatisfied	168	268	218	104	219	105

*Note.* ACC = Accident Compensation Corporation; NISS = New Injury Severity Score. Column totals for each variable vary, as missing values have not been reported

Table 3 presents the results of univariate analyses estimating the relative risk of distress at 12 months and 24 months post-injury across the different pre-injury, injury, health service-related, and early post-injury characteristics. A range of variables were associated with an increased risk of distress at 12 months. Most relationships were also observed with respect to distress at 24 months, with the exception of living with family or a partner no longer being associated with reduced risk of distress. In addition, individuals identifying Asian ethnicities, those reporting hazardous pre-injury alcohol consumption, or those who had a bad experience with ACC were also at increased risk of distress at 24 months.

**Table 3:** Univariate associations between sociodemographic and injury-related characteristics and distress at 12 and 24-months

	Distress at 12 Months			Distress at 24 Months		
	RR	95% CI for RR	*P* value	RR	95% CI for RR	*P* value
Pre-Injury Characteristics						
*Sex*						
Male	Ref			Ref		
Female	1.19	0.97, 1.45	0.09	1.17	0.97, 1.42	0.11
*Age (Years)*						
10–44	Ref			Ref		
45–65	1.18	0.97, 1.44	0.10	0.95	0.79, 1.16	0.63
*Ethnicity (Prioritised)*						
European	Ref			Ref		
Māori	1.91	1.51, 2.41		1.57	1.22, 2.00	
Pacific	1.92	1.35, 2.75		2.08	1.50, 2.89	
Asian	1.12	0.74, 1.69		1.88	1.36, 2.58	
Other	1.23	0.88, 1.72	< 0.01	1.32	0.97, 1.80	< 0.01
*Education*						
Less than secondary school	Ref			Ref		
Secondary school or higher	0.59	0.48, 0.72	< 0.01	0.69	0.56, 0.84	< 0.01
*Living Arrangements*						
Alone/With non-family	Ref			Ref		
With family	0.78	0.62, 1.00	0.05	0.90	0.71, 1.14	0.39
*Income Adequacy*						
Adequate	Ref			Ref		
Inadequate	2.08	1.71–2.54	< 0.01	2.11	1.74–2.55	< 0.01
*Working For Pay*						
No	Ref			Ref		
Yes	0.79	0.57, 1.09	0.16	0.77	0.56, 1.06	0.11
*Mental Health Conditions*						
0	Ref			Ref		
1	2.57	2.00, 3.30		2.00	1.52, 2.63	
2+	2.82	2.09, 3.79	< 0.01	3.11	2.38, 4.06	< 0.01
*Physical Health Conditions*						
0	Ref			Ref		
1	1.14	0.89, 1.45		1.12	0.89, 1.41	
2+	1.88	1.49, 2.38	< 0.01	1.58	1.24, 2.00	< 0.01
*General Health*						
Excellent/Very good	Ref			Ref		
Good	1.66	1.34, 2.07		1.58	1.28, 1.59	
Fair/poor	3.16	2.41, 4.16	< 0.01	2.86	2.18, 3.74	< 0.01
*Alcohol Use*						
Non-hazardous	Ref			Ref		
Hazardous	0.97	0.79, 1.19	0.76	0.82	0.68, 1.00	0.05
Injury-Related Characteristics						
*Injury Severity (NISS)*						
1–3 (Least severe)	Ref			Ref		
4–6 (Severe)	0.72	0.58, 0.89		0.79	0.65, 0.97	
7+ (Most Severe)	1.07	0.79, 1.45	< 0.01	0.84	0.61, 1.17	0.07
*Cause of Injury*						
Unintentional	Ref			Ref		
Intentional (Assault)	2.65	1.95, 3.60	< 0.01	1.97	1.38, 2.81	< 0.01
*Perceived Threat to Life*						
No	Ref			Ref		
Yes	2.10	1.66, 2.65	< 0.01	1.85	1.47, 2.34	< 0.01
Health Service Characteristics						
*Health Service Experience*						
Very good/Good/Moderate	Ref			Ref		
Bad/Very bad	1.64	1.07, 2.51	0.02	2.06	1.46, 2.91	< 0.01
*ACC Experience*						
Very good/Good/Moderate	Ref			Ref		
Bad/Very bad	1.21	0.82, 1.80	0.33	1.49	1.07, 2.09	0.02
*Access to Health Services*						
No Trouble	Ref			Ref		
Trouble/Mixed	1.54	1.16, 2.03	< 0.01	1.56	1.20, 2.03	< 0.01
Early Post-Injury Characteristics						
*Distress at 3 Months (K6 ≥ 8)*						
Not Distressed	Ref			Ref		
Distressed	4.54	3.73, 5.53	< 0.01	3.80	3.15, 4.58	< 0.01
*Expectations for Recovery*						
Already Recovered	Ref			Ref		
Expect to Recover Soon/Slowly	1.51	1.01, 2.05		1.42	1.06, 1.91	
Unsure	2.59	1.85, 3.63		2.50	1.81, 3.44	
Expect to Never Recover	2.56	1.57, 4.18	< 0.01	1.92	1.14, 3.23	< 0.01
*Satisfaction with Social Relationships*						
Satisfied	Ref			Ref		
Neutral/ Dissatisfied	2.70	2.22, 3.30	< 0.01	2.50	2.05, 3.04	< 0.01

*Note.* ACC = Accident Compensation Corporation; NISS = New Injury Severity Score; RR = Relative Risk

Table 4 presents data from two multivariable models, identifying significant predictors of distress at 12 months and 24 months respectively. Variables retained in the 12 month model included age, ethnicity, income adequacy, pre-injury mental health conditions, general health, injury severity, health service access, and distress at 3 months post-injury. Those aged 45 and over at the time of their injury were at increased risk of distress 12 months later compared with those who were under 45 years of age, as were those who identified as Māori or Pacific compared with individuals of NZ European ethnicity. Participants who reported inadequate pre-injury income were at increased risk of distress 12 months post-injury compared to those with adequate income. Similarly, those reporting one or more mental health conditions prior to their injury were at increased risk of distress at this time point, as were those reporting ‘good’, ‘fair’, or ‘poor’ pre-injury health compared to individuals who perceived their pre-injury health to be ‘excellent’ or ‘very good’. Participants who experienced trouble accessing healthcare were at increased risk of distress compared to those who did not have trouble. Individuals with an NISS of 4–6 (representing severe injury) were at less risk of reporting distress at the 12-month interview than individuals who had an NISS of 1–3 (representing less severe injury); those with NISS> 6 (the most severely injured category) were not at lesser risk compared to those with NISS of 1–3. Those whose injury was due to assault were at increased risk of distress compared to those whose injury was accidental. Participants who reported clinically relevant distress scores (K6 ≥ 8) at the 3-month interview were also at elevated risk of reporting distress again 12 months after their injury. When restricting the 12 month model to complete cases (i.e. those who completed interviews at all three time points; *n* = 1852), all relationships remained significant with two exceptions: individuals of Pacific ethnicity were no longer at increased risk of distress relative to NZ Europeans and participants with an NISS of 4–6 were no longer at reduced risk of distress compared to those with an NISS of 1–3.

**Table 4:** Multi-variable models identifying predictors of distress at 12 months and 24 months post-injury

	Distress at 12 Months (*n* = 2094)			Distress at 24 Months (*n* = 2085)		
	RR	95% CI for RR	*P* value	RR	95% CI for RR	*P* value
Pre-Injury Characteristics						
*Age (Years)*						
10–44	Ref					
45–65	1.27	1.05, 1.53	0.02			
*Ethnicity*						
European	Ref			Ref		
Māori	1.70	1.35, 2.13		1.34	1.06, 1.70	
Pacific	1.64	1.15, 2.34		1.84	1.32, 2.57	
Asian	1.05	0.71, 1.57		1.92	1.42, 2.60	
Other	1.14	0.83, 1.57	< 0.01	1.21	0.89, 1.63	< 0.01
*Income Adequacy*						
Adequate	Ref			Ref		
Inadequate	1.59	1.30, 1.95	< 0.01	1.62	1.34, 1.96	< 0.01
*Mental Health Conditions*						
0	Ref			Ref		
1	1.72	1.33, 2.23		1.59	1.20, 2.11	
2+	1.49	1.10, 2.00	< 0.01	1.73	1.30, 2.30	< 0.01
*General Health*						
Excellent/Very good	Ref			Ref		
Good	1.33	1.08, 1.65		1.27	1.03, 1.56	
Fair/poor	1.67	1.24, 2.24	< 0.01	1.65	1.21, 2.23	< 0.01
Injury-Related Characteristics						
*Injury Severity (NISS)*						
1–3 (Least severe)	Ref					
4–6 (Severe)	0.78	0.64, 0.96				
7+ (Most Severe)	1.06	0.79, 1.43	0.03			
*Cause of Injury*						
Unintentional	Ref					
Intentional (Assault)	1.55	1.09, 2.21	0.02			
Health Service Characteristics						
*Access to Health Services*						
No Trouble	Ref			Ref		
Trouble/Mixed	1.40	1.09, 1.81	0.01	1.42	1.10, 1.83	0.01
Early Post-Injury Characteristics						
*Distress at 3 Months (K6 ≥ 8)*						
Not Distressed	Ref			Ref		
Distressed	3.47	2.80, 4.29	< 0.01	3.14	2.57, 3.83	< 0.01

The variables predictive of distress at 12 months continued to predict distress at 24 months post-injury, with the exception of age, injury severity, and injury cause, which were not retained in the 24-month model. Individuals of Asian ethnicity were also at increased risk of distress at 24 months compared with individuals of NZ European ethnicity. Explanatory variables in the 24-month model did not change when restricting the analysis to individuals who had participated in all three POIS interviews (*n* = 1852).

## Discussion

Clinically relevant distress was reported by 25% of a large cohort of injured New Zealanders, approximately 3 months post-injury. This had reduced to 15% by 12 months post-injury and 16% 24 months post-injury. Age, ethnicity, adequacy of income, pre-injury mental health conditions, pre-injury health status, accessibility of health services, injury cause and severity, and distress 3 months post-injury were significantly associated with distress 12 months post-injury. The majority of these associations were evident with respect to distress 24 months post-injury, with the exception of age, injury severity, and injury cause; distress at 3 months was most strongly associated with subsequent distress. To the best of our knowledge, this is the first investigation to identify factors predictive of distress in a general injury population up to 24 months after injury, either in NZ or internationally.

Although POIS participants had a diverse range of injury types and severities, including those typically classified as being of ‘minor’ threat to life, the prevalence of distress detected early after injury was high. Distress levels were comparable to those identified among patients hospitalised for injury in the Netherlands, where 23% of patients reported distress one week after injury and 14% of patients continued to report distress 12 months later (de Munter et al. 2020). Their study included individuals who had been admitted because of self-harm and used an alternative measure of distress (the Hospital Anxiety and Depression Scale) (Zigmond and Snaith 1983). It is likely that higher rates of distress would have been identified had individuals with injuries due to self-harm been included in POIS. This, or injury severity, may explain why distress among POIS participants was lower than the prevalence of distress and psychiatric disorder reported among trauma centre patients in Australia up to 12 months post-injury (Bryant et al. 2010; O’Donnell et al. 2004; Wiseman et al. 2015) and male emergency department patients in the United Kingdom up to 18 months post-injury (Mason et al. 2002).

While distress among POIS participants was lower than some studies focused on individuals with severe injuries, distress was higher than that reported in a sample of 248 individuals who experienced a minor injury in the United States (Richmond et al. 2009; Richmond et al. 2014). In that study, approximately 18% of the participants were diagnosed with depression in the 12 months after their injury (Richmond et al. 2009; Richmond et al. 2014). However, the Structured Clinical Interview for DSM IV-TR disorders was used to detect psychiatric disorder; such structured clinical interviews can result in more conservative estimates of distress prevalence and are typically used to identify serious mental illness (Pratt 2009). Despite the high prevalence of distress among POIS participants at 3 months post-injury, this prevalence is comparable to the level of distress in the general NZ population (22%), as indicated by responses to the K6 from 4401 adults who participated in The New Zealand Attitudes and Values Study (NZAVS) (Krynen et al. 2013). This is not surprising given that POIS participants were of working age at the time of their injury, and perceptions of their own health have previously been identified as higher than those of the general population (Wilson et al. 2014).

Several pre-injury sociodemographic characteristics were independently associated with an increased risk of distress at 12 months post-injury, including being over 45 years of age and identifying Māori or Pacific ethnicities. Asian ethnicity was also associated with greater distress at 24 months post-injury. Disparities in K6 distress scores across ethnic groups have previously been documented in NZ. The NZAVS found that Pacific and Asian peoples had the highest psychological distress levels, closely followed by Māori, while Pākehā/European participants had the lowest levels of distress (Krynen et al. 2013). Disparities are likely attributable to a complex range of historical, socioeconomic, and lifestyle factors (Ellison-Loschmann and Pearce 2006); the process and consequences of colonisation (for Māori) (Robson and Harris 2007); differences in opportunities to access culturally appropriate healthcare (Kapeli et al. 2020); and discrimination (Houkamau et al. 2017; Kapeli et al. 2020; Krynen et al. 2013).

In NZ, the ACC scheme provides up to 80% of an individual’s weekly income if they are unable to work due to an injury. The scheme also assists with treatment costs, with the amount paid for each type of treatment set by legislation and subject to change each year (Accident Compensation Corporation 2020). Our findings demonstrating that individuals reporting inadequate pre-injury income are at increased risk of distress at 12 and 24 months post-injury compared to those with adequate pre-injury income suggests that the scheme is not providing sufficient support for injured people. It is likely that reduced income following injury and the significant co-payments required to access healthcare and rehabilitation contribute to substantial distress among people already struggling to meet basic needs for accommodation, food, and other necessities (Jatrana and Crampton 2009). Consistent with our findings, responses to the 2003–2004 Te Rau Hinengaro (NZ Mental Health Survey) revealed that people with lower household income had higher levels of psychological distress (Oakley Browne et al. 2010).

Despite POIS participants having accessed ACC (and therefore having at least some contact and support from health services for their injury), a proportion of participants reported trouble accessing healthcare. This trouble independently predicted experiencing clinically relevant distress 12 and 24 months after injury. Separate POIS analyses have consistently identified trouble accessing healthcare as a predictor of subsequent disability (e.g. Derrett et al. 2013) and poor self-rated health (Langley et al. 2011), with these adverse relationships particularly pronounced for Māori (Wyeth et al. 2019). While cost undoubtedly represents a barrier to healthcare (Goodyear-Smith and Ashton 2019), other factors that may play a role include the distance or time needed to reach health services and previous negative experiences with healthcare.

Although injury cause was significantly associated with distress at 12 months, this relationship was no longer observed at 24 months. Furthermore, relationships between injury severity and distress were inconsistent and not detected at 24 month follow-up. Other studies have found injury cause, but not injury severity, to be associated with distress (Chiu et al. 2011). Among 210 male patients admitted to hospital for an injury, no relationship between injury severity and psychological status 18 months post-injury was found (Mason et al. 2002). It is likely that individual perceptions have a stronger relationship with distress than measures of severity based on short-term threat to life. In a study of 426 patients hospitalised for injury in the United States, self-reported perceived injury severity (which had no correlation with injury severity scores) significantly predicted decreased physical and mental quality of life six months later (Brasel et al. 2010).

Those who sustained their injury because of an assault were at increased risk of distress at 12 months post-injury compared to those experiencing unintentional injury. This risk was no longer evident at 24 months post-injury, although further research is required to confirm this finding. While available studies of general injury populations have not examined predictors of distress up to 24 months after an injury, several have shown violent injury and prior lifetime trauma exposure to be associated with an increased risk of subsequent depressive symptoms and post-traumatic stress disorder (PTSD), even after adjustment for demographic factors (Chiu et al. 2011; Rahtz et al. 2017; Shalev et al. 2019).

The experience of pre-existing mental health conditions and lower levels of self-rated general health were also associated with increased distress at follow-up. This is consistent with findings from 4239 patients admitted to hospital for injury in the Netherlands, where pre-injury frailty and psychological complaints were important prognostic factors for psychological distress at 12 months after injury (de Munter et al. 2020). Research involving smaller samples of people hospitalised for injury has also identified pre-existing psychiatric conditions as an independent predictor of psychological distress at 12 months (Skogstad et al. 2014) and 18 months (Mason et al. 2002) post-injury, respectively.

Other studies have focused on the role of early post-injury distress and note that greater distress early after injury is predictive of elevated future distress (Richmond and Kauder 2000; Shalev et al. 2019), as was the case for POIS participants. For example, depression and post-traumatic stress symptoms at time of hospital admission were the only significant predictors of PTSD and global distress in the first 6 months post-injury among 63 patients admitted to a major trauma centre in the United Kingdom (Johnson et al. 2019). In Australia, an investigation of 201 patients hospitalised for injury found that along with intensive care unit admission, and high levels of depression, anxiety and stress at 3 months post-injury predicted high levels at 6 months post-injury (Wiseman et al. 2015). Importantly, our findings reveal that the experience of pre- and early post-injury psychological distress is just as important to consider among those with injuries that do not necessitate hospitalisation.

### Limitations

While POIS participants are representative of working-age adults who have sustained injuries in NZ, the extent to which findings can be generalised to those with a broad range of injuries in other countries is unclear. Furthermore, although participant drop-out was low relative to many prospective cohort studies of this duration, it is important to acknowledge that there may be important differences between those who participated in all study interviews and those who did not (Langley et al. 2013). This may have resulted in an under or over-estimate of distress, depending on whether participants experiencing greater distress were more or less likely to complete a follow-up interview. It is also important to acknowledge that analyses were not restricted to participants with data at all three interview points but to those with complete data at each time point (in order to maximise statistical power). However, this did not result in substantial changes to the identified predictors of distress, with the exception of injury severity. The paper is reporting data collected a decade ago. Although there have been no major national health system changes during this time, is possible that specific services provided to support injured New Zealanders may have changed. The relationship between ethnicity and distress among POIS participants was examined using the prioritisation approach where those reporting multiple ethnicities are counted in a single (prioritised) category. Although this is a common practice in NZ, prioritisation does not allow for all self-reported ethnicities to be acknowledged and included in analyses (Didham and Callister 2012). Finally, the absence of information on pre-injury psychological distress is a limitation as this may be an important contributor to distress experienced in response to injury and subsequent distress. Nevertheless, early identification of distress symptoms following injury, and subsequent preventive intervention, may reduce long-term distress symptoms and improve recovery from injury.

### Clinical implications

Our findings have clear implications for health professionals who provide treatment and rehabilitation for injured people. Approximately 25% of those they are caring for can be expected to experience clinically relevant distress in the first 3 months after their injury, regardless of injury severity. Given that early post-injury distress was the strongest predictor of subsequent distress, screening for distress as early as possible in the injury care pathway is important, as has been previously advocated (O'Donnell et al. 2008).

There are a number of brief tools that can be used to efficiently and accurately identify individuals in need of psychological support, such as the K6 (Kessler et al. 2002). Research has demonstrated the feasibility of systematic screening for PTSD and depression among people attending the emergency department for a serious injury (Jaramillo et al. 2019). Future studies are needed to explore the utility of distress screening in additional settings, such as general practices and physiotherapy clinics. Screening in a range of settings would enable timely referral to mental health professionals for those in need. However, research is also needed to examine whether the entire pathway of screening through to psychological intervention is effective at reducing distress among injured populations (Perkes et al. 2014). Professional support to manage the psychosocial difficulties resulting from injury is strongly desired, particularly by individuals recovering from serious injury (Brand et al. 2018).

## Conclusions

The experience of clinically relevant distress is common after injury, including after injuries traditionally considered to be minor. Our investigation has identified factors that confer a greater risk of long-term psychological distress among injured people, with levels of distress early post-injury warranting particular attention from health professionals. Screening for distress is necessary to facilitate early psychological intervention and promote long-term recovery from injury (Jacoby, Shults, & Richmond, 2017). Given the incidence of injury each year, distress screening and referral to treatment in the injured population may also help to reduce the high number of people affected by mental illness and distress in NZ, and remove barriers to greatly needed mental healthcare (New Zealand Government 2018).

## Data Availability

The data collected and analysed in this study cannot be shared due to ethical constraints.
